# Effects of Physical Exercise on the Microbiota in Irritable Bowel Syndrome

**DOI:** 10.3390/nu16162657

**Published:** 2024-08-11

**Authors:** Chunpeng Li, Jianmin Li, Qiaorui Zhou, Can Wang, Jiahui Hu, Chang Liu

**Affiliations:** 1 Russian Sports University, Moscow 105122, Russia; lichunpeng61@gmail.com; 2 School of Tai Chi Culture Handan University, Handan 056005, China; dimitryheider927@gmail.com; 3 College of Food Science & Nutritional Engineering, China Agricultural University, Beijing 100083, China; 2021306120130@cau.edu.cn (Q.Z.); b20203060495@cau.edu.cn (C.W.); 4 Moscow State Normal University, Moscow 127051, Russia; 5 School of Sport Science, Beijing Sport University, Beijing 100084, China

**Keywords:** athletes, microbiome, immune function, infection risk, athletic performance

## Abstract

Irritable bowel syndrome (IBS) is a prevalent functional gastrointestinal disorder characterized by abdominal pain, bloating, diarrhea, and constipation. Recent studies have underscored the significant role of the gut microbiota in the pathogenesis of IBS. Physical exercise, as a non-pharmacological intervention, has been proposed to alleviate IBS symptoms by modulating the gut microbiota. Aerobic exercise, such as running, swimming, and cycling, has been shown to enhance the diversity and abundance of beneficial gut bacteria, including Lactobacillus and Bifidobacterium. These bacteria produce short-chain fatty acids that possess anti-inflammatory properties and support gut barrier integrity. Studies involving IBS patients participating in structured aerobic exercise programs have reported significant improvements in their gut microbiota’s composition and diversity, alongside an alleviation of symptoms like abdominal pain and bloating. Additionally, exercise positively influences mental health by reducing stress and improving mood, which can further relieve IBS symptoms via the gut–brain axis. Long-term exercise interventions provide sustained benefits, maintaining the gut microbiota’s diversity and stability, supporting immune functions, and reducing systemic inflammation. However, exercise programs must be tailored to individual needs to avoid exacerbating IBS symptoms. Personalized exercise plans starting with low-to-moderate intensity and gradually increasing in intensity can maximize the benefits and minimize risks. This review examines the impact of various types and intensities of physical exercise on the gut microbiota in IBS patients, highlighting the need for further studies to explore optimal exercise protocols. Future research should include larger sample sizes, longer follow-up periods, and examine the synergistic effects of exercise and other lifestyle modifications. Integrating physical exercise into comprehensive IBS management plans can enhance symptom control and improve patients’ quality of life.

## 1. Introduction

Irritable bowel syndrome (IBS) is a prevalent functional gastrointestinal disorder affecting a significant portion of the global population [1]. It is characterized by a range of symptoms including abdominal pain, bloating, diarrhea, and constipation [2]. These symptoms can vary widely among individuals and can fluctuate in severity, leading to a substantial impact on the quality of life [3]. IBS can negatively affect a person’s daily activity, work, leisure time, sleep, eating habits, ability to travel, sexual function, and work-related roles. According to the International Foundation for Functional Gastrointestinal Disorders (IFFGD), more than two-thirds of respondents (68 per cent) report experiencing these above-mentioned impacts [4].

A study indicates that IBS occurs via a variety of pathophysiological mechanisms, including altered gastrointestinal motility, visceral hypersensitivity, altered intestinal permeability, immune activation, enteroencephalic dysregulation, central nervous system dysfunction, and alterations in the intestinal microbiota [4]. Although the exact cause of IBS remains elusive, it is generally understood to involve a complex interplay of factors, including abnormalities in gut motility, visceral hypersensitivity, altered mucosal and immune function, gut–brain interactions, and, notably, the gut microbiota [5].

The Role of the Gut Microbiota in IBS: Recent advances in microbiome research have highlighted the crucial role of the gut microbiota in maintaining gastrointestinal health and its potential involvement in the pathogenesis of IBS [6]. The gut microbiota consists of trillions of microorganisms, including bacteria, viruses, fungi, and archaea, which coexist symbiotically within the human intestines [7]. These microorganisms are essential for various physiological processes, such as digestion, immune function, and the synthesis of essential nutrients [8].

Normally, commensal microorganisms in the gut regulate signaling molecules and metabolites that are essential for the maintenance of intestinal homeostasis and the development of the mucosal immune system. When the intestinal biome is disturbed, it leads to inflammation, triggering oxidative stress and increased intestinal permeability, which may result in changes in the location of bacteria on the mucosal surface [9]. In IBS patients, the composition and function of the gut microbiota are often disrupted, a condition known as dysbiosis [10].

Dysbiosis in IBS is characterized by reduced microbial diversity, an imbalance between beneficial and pathogenic bacteria, and alterations in microbial metabolites such as short-chain fatty acids (SCFAs) [11]. These changes can contribute to the symptoms of IBS through several mechanisms, including increased intestinal permeability, altered gut motility, and heightened visceral sensitivity [12]. Moreover, the gut–brain axis, a bidirectional communication system between the central nervous system and the gastrointestinal tract, plays a pivotal role in IBS [13]. Dysbiosis can affect the gut–brain axis, leading to an exacerbation of gastrointestinal symptoms and associated psychological conditions such as anxiety and depression [14].

Non-Pharmacological Interventions for IBS: Given the multifactorial nature of IBS, treatment approaches are often tailored to address individual symptoms and underlying causes. Traditional treatments for IBS include dietary modifications, pharmacotherapy, and psychological interventions [15]. However, these treatments are not always effective for all patients and may come with undesirable side effects [16]. For example, anti-diarrheal drugs and laxatives are used to treat diarrhea and constipation, respectively, but they are not effective in the treatment of abdominal pain, and currently available treatment options can only alleviate symptoms in a subset of patients. Psychological interventions, such as low-dose neuromodulatory drugs, can control central symptoms and regulate the gastrointestinal tract but may produce side effects [17]. Consequently, there is growing interest in non-pharmacological interventions that can offer holistic benefits without adverse effects [18].

Physical exercise is emerging as a promising non-pharmacological intervention for IBS [19]. Exercise has long been recognized for its wide-ranging health benefits, including improved cardiovascular health, enhanced immune function, and better mental health outcomes [20]. In the context of IBS, physical exercise is hypothesized to exert beneficial effects by modulating the gut microbiota, reducing systemic inflammation, and improving gut motility and psychological well-being [21].

Physical Exercise and Gut Microbiota Modulation: Physical exercise influences the gut microbiota through multiple mechanisms [22]. It can alter the gut environment, promote the growth of beneficial bacteria, and enhance microbial diversity [23]. Regular exercise has been shown to increase the production of SCFAs, which have anti-inflammatory properties and can strengthen the intestinal barrier [24]. The exact mechanism by which exercise induces an increase in the concentrations of fecal SCFAs is not clear, but it may involve promoting the production of SCFAs through an increase in endogenous metabolites (e.g., lactic acid), the increased mixing of intestinal contents, and the bacterial fermentation of dietary fibers due to an increase in anaerobic fermentation as a result of changes in colonic oxygen saturation or pH or a decrease in the intestinal utilization and uptake of SCFAs. Exercise-induced increases in fecal SCFAs may also be increased by changes in the functional capacity of the gut microbiota to produce SCFAs [25]. Additionally, exercise can reduce the levels of pro-inflammatory cytokines, thereby decreasing gut inflammation and improving overall gut health [26].

The type, intensity, and duration of exercise play significant roles in determining its effects on the gut microbiota [23]. Aerobic exercises, such as running, swimming, and cycling, are particularly effective in enhancing microbial diversity and promoting the growth of beneficial bacteria like Lactobacillus and Bifidobacterium [27]. Resistance training, which includes weight lifting and other strength-based activities, also has positive effects on the gut microbiota, although its mechanisms may differ from those of aerobic exercise [28]. Combined training programs that incorporate both aerobic and resistance exercises may offer synergistic benefits, leading to more comprehensive improvements in the gut microbiota’s composition and function [29].

The Clinical Evidence of Exercise Benefits in IBS: Several clinical studies have investigated the effects of physical exercise on IBS symptoms and gut microbiota composition [30]. These studies generally support the hypothesis that exercise can improve IBS symptoms by modulating the gut microbiota [31]. For instance, randomized controlled trials have shown that regular aerobic exercise can lead to significant reductions in abdominal pain, bloating, and bowel irregularities in IBS patients [30]. These improvements are often accompanied by positive changes in the gut microbiota, such as increased microbial diversity and higher levels of beneficial bacteria [32].

Moreover, exercise interventions have been found to improve mental health outcomes in IBS patients, which is crucial given the strong association between IBS and psychological conditions like anxiety and depression [33]. By enhancing mood and reducing stress, exercise can positively influence the gut–brain axis, further alleviating IBS symptoms [34].

Long-term Effects and Safety Considerations: While the short-term benefits of exercise for IBS patients are well-documented, the long-term effects and safety of sustained exercise interventions warrant further investigation [35]. Long-term exercise regimens are likely to provide ongoing benefits by maintaining gut microbiota diversity and reducing systemic inflammation [36]. However, it is important to ensure that exercise programs are tailored to individual capabilities and they need to avoid potential adverse effects.

Excessive or inappropriate exercise can exacerbate IBS symptoms and lead to increased intestinal permeability and gut dysbiosis [37]. Therefore, personalized exercise plans that start with low-to-moderate intensity and gradually increase in intensity and duration are recommended. Monitoring patient responses and adjusting exercise protocols as needed can help maximize the benefits while minimizing the risks.

Taken together, IBS is a multifaceted disorder that significantly impacts patients’ quality of life. The gut microbiota plays a crucial role in the pathogenesis of IBS, and modulating the microbiota through non-pharmacological interventions such as physical exercise is a promising approach to symptom management [38]. Various types and intensities of exercise can positively influence the gut microbiota, thereby improving IBS symptoms and overall health [22]. Although further research is needed to fully understand the long-term effects and safety of exercise interventions, current evidence supports the inclusion of physical exercise in a comprehensive IBS management strategy. This review aims to explore in detail the relationship between physical exercise, the gut microbiota, and IBS, providing a comprehensive overview of the potential for exercise to relieve and treat this challenging condition.

**Figure 1:**
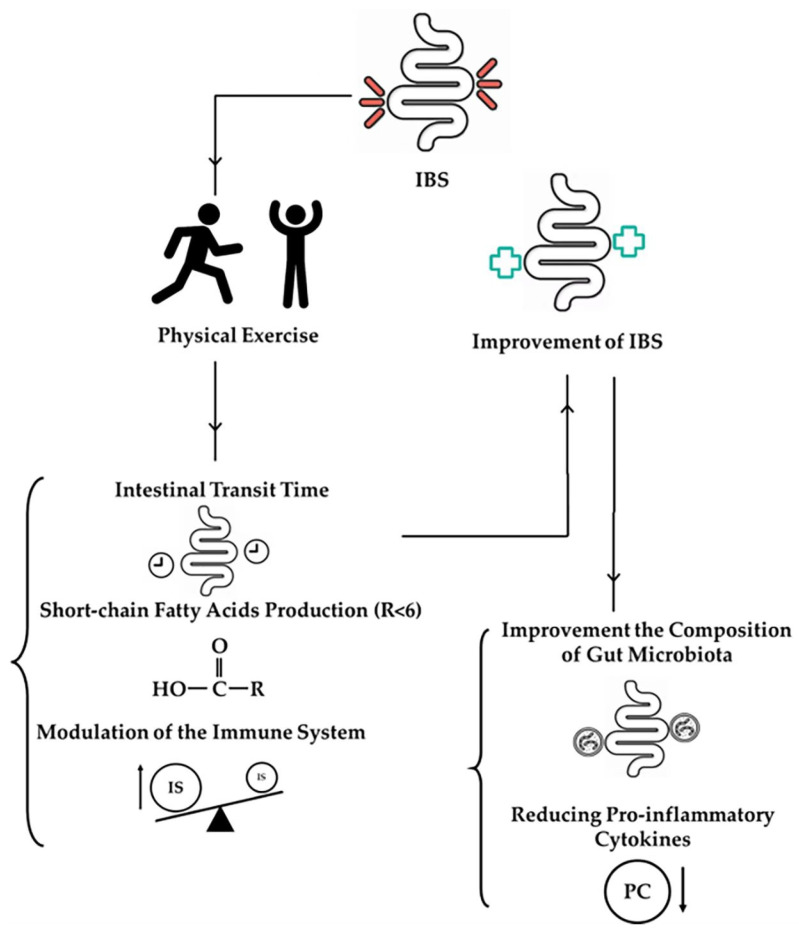
Effects of physical exercise on the gut microbiota in irritable bowel syndrome and on symptoms. IBS—irritable bowel syndrome; IS—immune system; PC—pro-inflammatory cytokines.

By synthesizing current research on the effects of physical exercise on the gut microbiota in IBS patients, this review will provide a comprehensive overview of how different types and intensities of exercise can influence gut health and alleviate IBS symptoms. Additionally, it will discuss the implications of these findings for developing effective, safe, and personalized exercise interventions for IBS patients. The goal is to highlight the potential of physical exercise as a non-pharmacological treatment option that can be integrated into broader IBS management plans to improve patients’ outcomes and quality of life (Figure 1).

## 2. Impact of Exercise on the Gut Microbiota in IBS Patients

### 2.1. Aerobic Exercise

Aerobic exercise, encompassing activities such as running, swimming, and cycling, is widely celebrated for its extensive health benefits [39]. These benefits include improvements in cardiovascular fitness, weight management, and mental well-being [40]. Physical exercise increases energy expenditure and reduces body weight in obese patients by depleting energy substances stored in the liver, muscle, and fat cells, including glycogen, fat, and protein, through the processes of glycolysis, the tricarboxylic acid cycle, and oxidative phosphorylation [41]

Additionally, during exercise, the undercarboxylated osteocalcin (ucOCN) gene is upregulated in the organism, which improves mental health by mediating the expression and signaling pathways of 5-hydroxytryptamine (5-HT), γ-hydroxybutyric acid (GABA), the hypothalamo-pituitary–adrenal axis (HPA axis), neurotrophic factors, and inflammatory responses [42], with eventual antidepressant results. Regular participation in aerobic exercise enhances heart and lung function, increases overall stamina, and helps maintain a healthy weight by burning calories and improving metabolic rate. Moreover, aerobic exercise has been shown to reduce symptoms of anxiety and depression, likely due to the release of endorphins and the reduction in stress hormones [43].

In addition to these well-known benefits, aerobic exercise exerts significant effects on gut microbiota composition, which can be particularly beneficial for patients with IBS [44]. Studies have demonstrated that aerobic exercise can increase the diversity of the gut microbiota, promoting the growth of beneficial bacteria such as Bifidobacterium and Lactobacillus [45]. For instance, patients with moderate to severe diarrhea-predominant IBS showed improvement in symptoms such as flatulence, stool pressure, and diarrhea after additional supplementation with Lactobacillus and Bifidobacterium compared to a placebo group [46], and these bacteria play crucial roles in maintaining gut health by producing SCFAs, which serve as energy sources for colon cells and help regulate immune responses; the details of the above content are shown below [47].

### 2.2. Resistance Exercise

Resistance exercise, or strength exercise, focuses on exercises that enhance muscle strength [48]. This form of exercise includes activities such as weight lifting, bodyweight exercises (e.g., push-ups, squats), and resistance band workouts [49]. Although research on the impact of resistance training on the gut microbiota is less extensive compared to aerobic exercise, preliminary studies suggest there are beneficial effects.

Resistance exercise may also contribute to gut health through its effects on the gut barrier’s function [28]. The gut barrier is crucial in preventing the translocation of harmful bacteria and toxins from the gut into the bloodstream [50]. A strong gut barrier helps maintain immune homeostasis and reduces the likelihood of chronic inflammation, which is often observed in IBS patients. Enhancing the integrity of the gut barrier through resistance exercise could, therefore, be a key mechanism by which this form of exercise benefits gut health [51].

### 2.3. Effects on Gut Microbiota Composition

Research indicates that regular aerobic exercise can enhance the diversity and abundance of beneficial gut bacteria [52]. For instance, habitual aerobic exercise has also been shown to significantly increase the populations of Lactobacillus and Bifidobacterium in the gut, which are crucial for gut health [53]. These beneficial bacteria produce SCFAs, such as butyrate, acetate, and propionate, which have anti-inflammatory properties and support the integrity of the gut barrier [45].

For example, a recent study has elucidated the changes in the intestinal flora induced by aerobic exercise training and its effects on the physiological adaptability of a person’s endurance exercise capacity. In this study, ICR mice were randomly divided into three groups: vehicle intake + sedentary (V+S), vehicle intake + exercise training (V+Ex), and antibiotic intake + exercise training (AB+Ex). The mice in the exercise training groups performed treadmill running for 8 weeks. During this period, the mice in the antibiotic intake group had free access to water containing antibiotics.

In a second study, ICR mice were randomly divided into three groups: a sham operation group, sedentary mouse cecal microbiota transplantation group (Sed-CMT), and exercise-trained mouse cecal microbiota transplantation group (Ex-CMT). In the first study, the time to exhaustion during treadmill running (a measure of maximum aerobic capacity) in the V+Ex group was significantly longer than in the V+S and AB+Ex groups. The citrate synthase (CS) activity and PGC-1α protein levels in the gastrocnemius muscle of the V+Ex group were significantly higher than in the V+S and AB+Ex groups. The bacterial families Erysipelothrixaceae and Alcaligenesaceae were positively correlated with the time to exhaustion on the treadmill [54].

In the second study, the time to exhaustion on the treadmill after transplantation in the Ex-CMT group was significantly higher than in the sham and Sed-CMT groups. Additionally, the CS activity and PGC-1α protein levels in the gastrocnemius muscle of the Ex-CMT group were significantly higher than in the sham and Sed-CMT groups. Therefore, the gut microbiota altered by aerobic exercise training may be associated with enhanced endurance and improved muscle mitochondrial energy metabolism.

Aerobic exercise training alters the composition of the gut microbiota, with Erysipelothrix and Alcaligenes being among the altered bacteria. The gut microbiota is associated with endurance performance and the levels of metabolic regulators in the skeletal muscle after aerobic exercise training. Continuous antibiotic treatment attenuated the increases in endurance performance, skeletal muscle citrate synthase activity, and PGC-1α levels induced by aerobic exercise training. The transplantation of gut microbiota from exercise-trained mice improved endurance performance and metabolic regulator levels in recipient skeletal muscle, even without aerobic exercise training [54].

### 2.4. Enhancement of Microbial Diversity

Aerobic exercise is associated with an increase in microbial diversity, which is a key indicator of a healthy gut microbiome [52]. A diverse microbiota is better equipped to resist pathogenic invasions and to maintain various physiological functions essential for gut health [55]. Studies have demonstrated that individuals who engage in regular aerobic exercise tend to have a more varied and balanced gut microbiome compared to sedentary individuals [44].

A recent systematic review examined the effects of exercise on the gut microbiota in humans and animal models, which included 32 exercise studies, comprising 19 human studies and 13 animal studies. These studies discussed microbiome outcomes such as diversity, taxonomic composition, or microbial metabolites seen during the intervention period. The review found that over 50% of the human studies reported no significant effect of exercise on microbial diversity. When exercise did have a significant impact, it typically increased the Shannon index, indicating enhanced microbial richness and evenness, irrespective of disease status. Exercise was also associated with changes in beta diversity indicators, although no consistent pattern was observed [56].

In contrast to human studies, a greater proportion of animal studies reported changes in microbial diversity, though without a clear trend, primarily due to the varied effects of aerobic exercise on diversity indicators. Regarding taxonomic composition, exercise generally led to a decrease in the *Firmicutes*/*Bacteroidetes* ratio in humans, with increases in the proportions of *Bacteroidetes* and *Roseburia*. In animal models, the abundance of *Coprococcus*, another producer of SCFAs, increased with exercise. Overall, exercise in animal models tended to boost SCFA producers [56].

With respect to metabolites, SCFAs were the ones most frequently measured. However, due to the limited number of studies on the effects of exercise on microbial-produced metabolites, including SCFAs, no clear patterns have emerged. The overall risk of bias in the studies was considered neutral. This comprehensive systematic review highlights that exercise may influence the gut microbiota, particularly through changes in taxonomic composition [56].

### 2.5. Increase in Beneficial Bacteria

The specific increase in beneficial bacteria such as Lactobacillus and Bifidobacterium can have several positive outcomes [57]. These bacteria are known for their role in fermenting dietary fibers into SCFAs [58]. Butyrate, one of the most studied SCFAs, is particularly important for colon health as it serves as the primary energy source for colonocytes, the cells lining the colon [59]. Additionally, butyrate has anti-inflammatory effects, promoting a healthy gut environment by inhibiting the activity of pro-inflammatory cytokines [60].

A 12-week, nonrandomized, controlled trial was conducted involving 32 sedentary women aged 65 years and older. Participants were divided into two groups to receive different exercise interventions: trunk muscle training (TM) and aerobic exercise training (AE). The AE program consisted of brisk walking at an intensity of ≥3 metabolic equivalents (METs). The composition of the gut microbiota in their stool samples was analyzed before and after the training period. Additionally, daily physical activity was monitored using accelerometers, trunk muscle strength was measured with the modified Claus-Weber (KW) test, and cardiorespiratory fitness was assessed using the 6 min walk test (6MWT) and the 6 min walk distance (6MWD) [61].

The results showed that KW test scores, as well as the distance covered during the 6MWT and 6MWD, improved in both groups. Notably, the relative abundance of gut *Bacteroidetes* increased significantly only in the AE group, particularly in participants who showed increased brisk walking time. Overall, the increase in gut *Bacteroidetes* following the exercise intervention was correlated with an improved performance in the 6MWT and 6MWD. In conclusion, aerobic exercise training aimed at increasing brisk walking time may enhance gut *Bacteroidetes* abundance and improve cardiorespiratory fitness in healthy older women [61].

### 2.6. Clinical Evidence in IBS Patients

Clinical trials have provided compelling evidence supporting the benefits of aerobic exercise for patients with IBS. One such trial investigated the effects of treadmill aerobic exercise on symptom severity and quality of life in women with mild to moderate IBS. The study recruited 20 women with mild to moderate IBS, who were randomly divided into two groups: a treadmill exercise group (10 participants) and a control group (10 participants). The treadmill group performed aerobic exercise on a treadmill for six weeks (three times per week for 30 min), while the control group continued with their usual daily activities [62].

The results demonstrated that after six weeks of treadmill aerobic exercise, the treadmill group experienced significant improvements in IBS symptom severity (*p* ≤ 0.001) and quality of life (*p* = 0.001) compared to the control group. Furthermore, within the treadmill group, both symptom severity and quality of life improved significantly post-intervention compared to pre-intervention (*p* ≤ 0.001). In contrast, the control group showed no significant differences in symptom severity or quality of life before and after the study (*p* > 0.05). In conclusion, this study highlights that treadmill aerobic exercise significantly reduces symptom severity and enhances the quality of life in women with mild to moderate IBS [62].

Another study from clinical trials demonstrated that walking, a popular form of moderate-intensity aerobic exercise, can improve psychological and gastrointestinal health and alleviate symptoms associated with irritable bowel syndrome such as flatulence, bloating, and intestinal disturbances. This study investigated the effects of a moderate-intensity aerobic exercise program on clinical and psychological parameters in patients with IBS. A total of 40 IBS patients (11 men and 29 women; mean age 51.9 ± 7.8 years) participated in a 12-week aerobic exercise program. Participants completed questionnaires assessing their gastrointestinal symptoms, psychological status, and quality of life (QoL) before and after the intervention. Additionally, field tests, anthropometric measurements, and bioimpedance assessments were performed [63].

The results confirmed a significant improvement in IBS symptoms following the aerobic exercise program. Bloating was the most common symptom, and it, along with abdominal pain, was significantly reduced after the intervention. Psychological and QoL questionnaires indicated decreases in anxiety, depression, somatization, and stress levels. There was a correlation between anxiety/depression and abdominal pain severity and between stress and bloating severity. In conclusion, moderate-intensity aerobic exercise has a positive effect on gastrointestinal symptoms and psychological health, complementing diet and psychological support as a non-pharmacological treatment for IBS. These findings underscore the importance of aerobic exercise as an alternative approach to the treatment of IBS [63].

Another clinical trial demonstrated that moderate-intensity aerobic exercise improves gastrointestinal health and alleviates symptoms of IBS. This study explored the effects of moderate-intensity aerobic exercise on physical capacity and IBS symptoms in 40 patients from southern Italy (11 males, 29 females; mean age 52.10 ± 7.72 years). The exercise program involved moderate-intensity aerobic exercise (60–75% of HRmax) for at least 180 min per week. Before and after the intervention, participants completed the IBS-SSS questionnaire to assess their IBS symptoms, reported their physical activity levels, and underwent field tests to evaluate their physical capacity, which was quantified as a Global Physical Capacity Score (GPCS). A control group of 38 subjects (21 males, 17 females; mean age 53.71 ± 7.27 years) without lower gastrointestinal symptoms served as the No IBS group. No significant differences were found between the IBS patients and the No IBS subjects, except for the symptom score, as expected [30].

After the exercise intervention, all participants experienced significant improvements in both their IBS symptoms and physical capacity. Higher physical capacity levels correlated with greater reductions in IBS symptomatology, especially when the GPCS reached above-average values. In conclusion, engaging in moderate-intensity aerobic exercise for at least 180 min per week positively impacts IBS symptoms and physical capacity. Monitoring the GPCS in IBS patients provides insights into the relationship between physical activity and symptom severity, aiding healthcare professionals in creating effective treatment plans [30].

### 2.7. Symptom Alleviation

The alleviation of IBS symptoms through aerobic exercise can be attributed to several factors [64]. Firstly, aerobic exercise has been shown to increase the population of beneficial bacteria and enhance microbial diversity in the gut [23]. This change in the gut microbiome promotes the production of SCFAs, which play crucial roles in maintaining gut health [65].

SCFAs serve multiple functions in the gut. They provide an energy source for colonocytes, the cells lining the colon, thereby supporting the integrity of the gut lining [66]. This maintenance of gut barrier function is essential in preventing leaky gut syndrome, which is often associated with IBS [67]. Furthermore, SCFAs have anti-inflammatory properties, with butyrate being particularly effective in reducing gut inflammation [47]. Chronic inflammation is also a common issue in IBS patients, contributing to symptoms such as abdominal pain, bloating, and irregular bowel movements [68].

Butyrate and other SCFAs modulate the immune response in the gut by inhibiting the production of pro-inflammatory cytokines and promoting the production of anti-inflammatory cytokines [69]. This modulation helps to balance the immune system and reduce the overall inflammatory state in the gut. By decreasing inflammation, aerobic exercise can help alleviate pain and discomfort, which are hallmark symptoms of IBS [63].

Additionally, regular aerobic exercise is known to enhance gut motility, which can be particularly beneficial for IBS patients [70]. Improved gut motility helps in the regular passage of stool, reducing constipation and preventing the buildup of gas and bloating [71]. This improvement in bowel regularity can lead to significant relief from IBS symptoms.

Moreover, aerobic exercise has systemic benefits that contribute to overall well-being and stress reduction [42]. Exercise stimulates the release of endorphins, which are natural painkillers and mood elevators [72]. This can help IBS patients manage stress, anxiety, and depression, which are often comorbid with IBS and can exacerbate its symptoms. By improving mental health and reducing stress, aerobic exercise indirectly supports gut health and symptom management [73].

In summary, the alleviation of IBS symptoms through aerobic exercise is multifaceted. It involves the enhancement of beneficial gut bacteria and microbial diversity, the production of anti-inflammatory SCFAs, the improvement of gut motility, and the reduction of stress and anxiety. These combined effects contribute to a significant decrease in the primary symptoms of IBS.

### 2.8. Reduction in Pro-Inflammatory Cytokines

In addition to changes in the gut microbiota, aerobic exercise has been associated with a decrease in pro-inflammatory cytokines [74]. Cytokines are small proteins that play a critical role in cell signaling, and an imbalance in these proteins can lead to chronic inflammation. Chronic inflammation is a known contributor to various gastrointestinal disorders, including IBS.

By reducing the levels of pro-inflammatory cytokines, aerobic exercise helps create an anti-inflammatory environment in the gut. This anti-inflammatory effect not only supports overall gut health but also alleviates IBS symptoms. Specifically, a reduction in cytokines such as tumor necrosis factor-alpha (TNF-α), interleukin-6 (IL-6), and interleukin-1 beta (IL-1β) can decrease the inflammatory response in the gut, leading to improved intestinal function and reduced discomfort associated with IBS [75].

Furthermore, the anti-inflammatory benefits of aerobic exercise extend beyond the gut, contributing to enhanced systemic health. Regular aerobic exercise promotes a balanced immune response, which is crucial for maintaining homeostasis and preventing excessive inflammatory reactions, which can exacerbate IBS symptoms. Overall, incorporating aerobic exercise into the management plan for IBS patients offers a multifaceted approach to improving gut health and reducing symptom severity.

## 3. Mechanisms Underlying Exercise-Induced Changes in the Gut Microbiota

### 3.1. Intestinal Transit Time

One of the primary mechanisms by which aerobic exercise influences the gut microbiota is through the modulation of intestinal transit time. Regular physical activity enhances gut motility, which is crucial for maintaining an optimal transit time for food and waste products through the digestive tract. An optimal transit time ensures that nutrients are adequately absorbed and that waste products are efficiently excreted [76]. This process prevents the overgrowth of pathogenic bacteria that can thrive in stagnant environments, thus promoting a balanced gut microbiome [77].

Enhanced gut motility resulting from aerobic exercise helps in reducing the time that food spends in the colon [78]. This reduction in transit time decreases the likelihood of harmful bacterial fermentation and the production of toxic metabolites [79]. Consequently, this environment supports the proliferation of the beneficial bacteria which are essential for maintaining gut health. These bacteria produce SCFAs, which serve as an energy source for colonocytes and have anti-inflammatory properties, further contributing to a healthy gut environment [66].

Additionally, aerobic exercise induces physiological changes that benefit the gut microbiota. For instance, increased blood flow to the digestive organs during physical activity enhances nutrient and oxygen delivery, which supports the growth and activity of beneficial microbes [80]. The anti-inflammatory effects of regular exercise also reduce systemic inflammation, which is linked to gut health. Lower inflammation levels can help maintain the integrity of the gut barrier, preventing conditions like leaky gut syndrome, where toxins and microbes pass through the intestinal wall into the bloodstream [81].

### 3.2. SCFA Production

As mentioned earlier, aerobic exercise boosts the production of SCFAs, which have numerous benefits for gut health. SCFAs are produced by the fermentation of dietary fibers by gut bacteria. These molecules play a critical role in maintaining gastrointestinal health and have systemic effects on the host.

Role of SCFAs in Gut Health: SCFAs serve as a primary energy source for colonocytes, the cells lining the colon. Butyrate, in particular, is vital for the health of colonocytes as it fuels their metabolism and supports cellular processes that maintain the integrity of the gut lining. By providing an energy source for these cells, SCFAs promote the repair and regeneration of the intestinal epithelium, enhancing its barrier function [82].

The anti-inflammatory properties of SCFAs are another crucial benefit. SCFAs inhibit the production of pro-inflammatory cytokines and promote the production of anti-inflammatory molecules. This modulation of the immune response helps in reducing intestinal inflammation, which is a common issue in IBS patients. Chronic inflammation can disrupt the gut barrier, leading to increased intestinal permeability, commonly referred to as “leaky gut”. By mitigating inflammation, SCFAs help maintain the integrity of the gut barrier [83].

Prevention of Translocation: By maintaining the integrity of the gut barrier, SCFAs prevent the translocation of harmful bacteria and toxins from the gut into the bloodstream. This translocation can trigger systemic inflammation and contribute to the development of various diseases, including metabolic disorders, liver diseases, and autoimmune conditions [84,85]. By enhancing the gut barrier’s function, SCFAs play a protective role in preventing these adverse health outcomes.

Systemic Effects of SCFAs: Beyond the gut, SCFAs have systemic effects that contribute to overall health. They influence the regulation of energy metabolism and have been shown to improve glucose homeostasis and lipid metabolism [86]. SCFAs can also cross the blood–brain barrier and affect brain function, potentially influencing mood and cognitive functions [87]. This is particularly relevant for IBS patients, who often experience comorbid psychological conditions such as anxiety and depression [88]. By improving gut health and reducing inflammation, SCFAs can have a positive impact on the gut–brain axis, alleviating both gastrointestinal and psychological symptoms associated with IBS [89].

Exercise and SCFA Production: Aerobic exercise has been shown to increase the production of SCFAs by promoting the growth of beneficial gut bacteria that are efficient at fermenting dietary fibers [90]. Studies have demonstrated that individuals who engage in regular aerobic exercise have higher levels of SCFAs in their gut compared to sedentary individuals [91]. This increase in SCFA production is associated with enhanced microbial diversity and a healthier gut microbiota profile [92,93].

The type and duration of exercise can influence the extent of SCFA production. Moderate-intensity aerobic exercises, such as brisk walking, cycling, and swimming, are particularly effective in promoting SCFA production [94]. Long-term adherence to an aerobic exercise regimen is essential for sustaining elevated levels of SCFAs and reaping long-term benefits for gut health and overall well-being [95].

Implications for IBS Management: For IBS patients, the enhancement of SCFA production through regular aerobic exercise can be a crucial component of symptom management. By increasing the levels of SCFAs, exercise helps to stabilize the gut environment, reduce inflammation, and strengthen the gut barrier. These effects can lead to a reduction in the severity of IBS symptoms, such as abdominal pain, bloating, and irregular bowel movements [96].

Moreover, the systemic benefits of SCFAs, including improved metabolic [97] and psychological health [98], provide additional support for incorporating aerobic exercise into comprehensive IBS treatment plans. Healthcare providers can recommend tailored exercise programs that focus on moderate-intensity aerobic activities to maximize the production of SCFAs and improve patient outcomes.

In summary, the production of SCFAs through aerobic exercise offers significant benefits for gut health, particularly for individuals with IBS. SCFAs support colonocyte energy metabolism, reduce inflammation, and maintain gut barrier integrity, preventing the translocation of harmful substances. The systemic effects of SCFAs further enhance overall health, making aerobic exercise a valuable non-pharmacological intervention for managing IBS. Future research should continue to explore the optimal exercise protocols for maximizing SCFA production and their long-term impacts on IBS and other gut-related disorders.

### 3.3. Modulation of the Immune System

Aerobic exercise also exerts significant effects on the immune system, which in turn influences gut health. Regular physical activity has been shown to modulate the immune response in several ways, reducing systemic inflammation and enhancing mucosal immunity [99,100]. This improved immune function helps maintain a healthy and balanced gut microbiota, which is essential for managing IBS symptoms [101].

One of the key ways aerobic exercise modulates the immune system is by reducing the levels of pro-inflammatory cytokines, such as TNF-alpha and IL-6, which are often elevated in chronic inflammatory conditions [102]. This reduction in systemic inflammation can alleviate the inflammatory milieu that negatively impacts the gut environment. By lowering these inflammatory markers, aerobic exercise creates a more favorable environment for beneficial gut bacteria, thereby supporting gut health [103].

In addition to reducing inflammation, aerobic exercise enhances mucosal immunity by increasing the production of immunoglobulin A (IgA) in the gut. Strenuous physical activity has been shown to increase sympathetic nervous system activity. Another rat-based study showed that both parasympathetic and sympathetic nerves promote IgA secretion [104]. IgA plays a crucial role in the immune system’s defense against pathogens by neutralizing harmful microbes and preventing them from adhering to the gut lining [105]. This enhanced mucosal immunity helps to maintain the integrity of the gut barrier, preventing pathogens from penetrating the intestinal wall and causing infections or triggering immune responses, which can lead to gut dysbiosis.

Furthermore, aerobic exercise promotes the circulation of immune cells, such as lymphocytes and natural killer cells, which are essential for detecting and eliminating pathogens [106]. This improved immune surveillance ensures that harmful bacteria are kept in check, allowing beneficial bacteria to thrive. Maintaining this balance between microbial populations is crucial for gut health, as an overgrowth of pathogenic bacteria can lead to conditions such as small intestinal bacterial overgrowth and IBS.

Another important aspect of exercise-induced immune modulation is the enhancement of the gut–brain axis [22,107]. Regular physical activity has been shown to reduce stress and anxiety, which can have a positive impact on gut health. Stress is known to negatively affect the gut microbiota and exacerbate IBS symptoms by increasing gut permeability and altering gut motility. By reducing stress levels, aerobic exercise helps to stabilize the gut environment, promoting a healthy microbiome.

In summary, the modulation of the immune system through aerobic exercise plays a vital role in supporting gut health. By reducing systemic inflammation, enhancing mucosal immunity, improving immune surveillance, and positively influencing the gut–brain axis, regular physical activity helps to maintain a balanced and healthy gut microbiota. These combined effects are particularly beneficial for individuals managing IBS symptoms, highlighting the importance of incorporating aerobic exercise into their routine.

## 4. Practical Implications, Recommendations, and Adaptive Strategies for IBS

### 4.1. Practical Implications for IBS Patients

Given the positive effects of aerobic exercise on the gut microbiota and IBS symptoms, incorporating regular aerobic exercise into the lifestyle of IBS patients can be highly beneficial. Healthcare providers should consider recommending structured aerobic exercise programs as part of a comprehensive treatment plan for IBS.

Implementing aerobic exercise into the daily routine of IBS patients can start with simple, low-impact activities such as walking, swimming, or cycling. These activities are generally well-tolerated and can be gradually increased in intensity and duration to suit the individual’s fitness level and tolerance. Starting with 20–30 min of moderate exercise, three to five times a week, can significantly impact gut health and overall well-being. For example, walking briskly in a local park, joining a community swim session, or participating in a cycling group can make exercise more enjoyable and sustainable.

Antonella Bianco et al. [30], from southern Italy, recruited patients with Celiac Disease and Functional Disorders to assess their physical indices (height, weight, body mass index, etc.) and performed physical assessment tests on them (including a 2 km walking experiment and grip strength and flexibility tests). The intervention program lasted 12 weeks, with moderate aerobic exercise carried out three times a week, and walking was used for exercise. The post-intervention assessment using the IBS Severity Assessment System showed that improving physical capacity and elevating physical activity can reduce IBS symptoms. This suggests that healthcare professionals, and teams with improvements and a treatment plan, can improve gastrointestinal symptoms in patients with IBS through moderate aerobic exercise for 180 min per week or more [30].

For patients new to exercise or those with severe symptoms, it may be helpful to consult with a physical therapist or a fitness professional who can design a personalized exercise plan that accommodates their specific needs and limitations. This approach ensures that the exercise regimen is both safe and effective, minimizing the risk of exacerbating symptoms. A tailored plan might include specific warm-up routines, flexibility exercises, and gradual progression schedules to build up endurance and strength without overburdening the patient.

In addition to the physical benefits, regular aerobic exercise can also provide psychological benefits, which are particularly important for IBS patients. Studies show that anxiety, depression, and IBS involve multiple gut microbial alterations. Additionally, research confirms that patients with both depression and IBS exhibit more pronounced alterations in their gut microbiota than those with only one disorder. Based on these findings, Carra A Simpson and his team at the University of Melbourne, Australia, aimed to characterize the microbial and physiological features of anxiety, depression, and IBS. They recruited four groups of female subjects: those with neither depression/anxiety nor IBS, those with IBS, those with depression/anxiety, and those with both depression/anxiety and IBS. The subjects filled out questionnaires and provided feces, urine, and saliva samples. These samples were tested for intestinal and oral biota and immune and endocrine markers, as well as undergoing a metabolomics analysis. This study informs the development of interventions for people with depression, anxiety, and/or IBS [108].

Exercise has been shown to reduce stress, anxiety, and depression, which are common comorbidities in IBS. By enhancing mood and reducing stress, aerobic exercise can help break the cycle of stress-induced symptom flare-ups, leading to more stable and manageable IBS symptoms. Mind–body exercises like yoga and Tai Chi can be particularly beneficial, as they combine physical activity with stress reduction techniques.

Furthermore, integrating aerobic exercise with other lifestyle modifications, such as dietary changes, adequate hydration, and proper sleep, can amplify its positive effects. For instance, combining exercise with a diet rich in fiber, probiotics, and prebiotics can synergistically enhance the gut microbiota’s diversity and function. Patients should be encouraged to maintain a balanced diet that supports gut health, avoiding known IBS triggers such as high-fat foods, caffeine, and certain fermentable oligosaccharides, disaccharides, monosaccharides, and polyols (FODMAPs) [109]. Nutritional counseling might be advantageous, helping patients to develop meal plans that support their exercise routines and digestive health.

Healthcare providers should also emphasize the importance of consistency and a long-term commitment to an exercise routine. While immediate benefits can be observed, the most significant improvements in gut health and symptom management are often observed over an extended period. Patients should be educated on setting realistic goals and expectations and celebrating small victories to stay motivated. Tools such as fitness trackers, exercise logs, and regular follow-up appointments can help patients stay on track and monitor their progress.

Support from healthcare professionals [110], family [111], and peer support groups can also play a crucial role in maintaining adherence to an exercise program. Sharing experiences and challenges with others who have similar conditions can provide motivation and practical tips for managing symptoms and staying active. Online forums, local support groups, and social media communities can offer a sense of camaraderie and encouragement, making the journey towards better health more engaging and less isolating.

In summary, the practical implications of incorporating aerobic exercise into the lifestyle of IBS patients are profound. Regular physical activity not only improves gut motility and microbiota balance but also reduces stress and enhances patients’ overall quality of life. Healthcare providers should advocate for structured, personalized aerobic exercise programs as an integral part of the holistic management of IBS, empowering patients to take an active role in their health and well-being. By fostering a supportive environment and providing comprehensive resources, healthcare providers can help IBS patients achieve better symptom control and an improved quality of life through regular aerobic exercise.

### 4.2. Exercise Recommendations

For optimal benefits, IBS patients are recommended to engage in moderate-to-vigorous aerobic exercise for at least 150 min per week. This recommendation aligns with guidelines from health authorities such as the American Heart Association and the Centers for Disease Control and Prevention, which has highlighted the broad health advantages of regular physical activity. Engaging in such a regimen can significantly improve gut motility, enhance immune function, and alleviate stress, all of which are crucial for managing IBS symptoms effectively.

Flexible Approaches to Achieving 150 Minutes: Achieving a 150 min weekly goal can be approached flexibly to suit individual preferences and schedules. This can be broken down into manageable sessions, such as 30 min of exercise five days a week, or even shorter, more frequent sessions that cumulatively reach the weekly target [112]. Flexibility in scheduling allows patients to fit exercise into their daily routines without feeling overwhelmed. This approach ensures consistency and helps integrate physical activity into being a natural part of their lifestyle [113].

Variety of Activities and Their Benefits: Various activities such as brisk walking, running, swimming, or cycling can be incorporated. Each of these activities offers unique benefits.

Brisk Walking: This is an accessible and low-impact exercise that can be easily integrated into daily routines. Walking in natural settings, such as parks or trails, can also provide additional mental health benefits by offering exposure to nature. Regular walking can enhance cardiovascular health and is gentle enough for beginners [114].

Running: For those seeking a higher intensity workout, running can significantly boost cardiovascular fitness and endorphin levels, contributing to improved mood and better stress management. Running can be tailored to different fitness levels by adjusting the pace and distance [115].

Swimming: As a full-body workout that is gentle on the joints, swimming is ideal for individuals with joint pain or injuries. Swimming also promotes relaxation and can serve as a soothing way to end the day. Swimming engages multiple muscle groups, enhancing overall body strength and endurance [116].

Cycling: Whether done outdoors or on a stationary bike, cycling provides a robust cardiovascular workout and can be an enjoyable way to explore new places or participate in virtual cycling classes. Cycling helps improve lower body strength and enhance cardiovascular health [117].

Ensuring Enjoyment and Sustainability: It is crucial for individuals to choose activities that they enjoy and can sustain in the long term to ensure adherence to their exercise regimen. Enjoyment and personal interest are key factors in maintaining motivation and consistency [118]. Patients might find it helpful to vary their activities to prevent boredom and address different fitness components. For instance, combining swimming and cycling can provide both upper and lower body workouts, enhancing overall fitness and engagement. Engaging in different forms of exercise also helps prevent overuse injuries and keeps the workout routine fresh and exciting [119].

Leveraging the Social Aspects of Exercise: Additionally, the social aspects of exercise can significantly enhance adherence to fitness routines. Joining local walking groups, running clubs, or fitness classes can provide a sense of community and support. Engaging in group activities not only makes exercise more enjoyable but also introduces a level of accountability, helping individuals stick to their routines. Social interactions during exercise can improve mental health and provide emotional support, which is particularly beneficial for individuals with IBS who may feel isolated due to their symptoms [120].

The Timing and Context of Exercise Routines: Patients should also consider the timing and context of their exercise routines to optimize their benefits. Some may prefer morning workouts to start the day with increased energy and an uplifted mood, while others may find evening exercise helps them unwind. Understanding how their body responds to exercise at different times can help patients tailor their schedules to their preferences and needs. Tailoring the timing of exercise to fit individual lifestyles can enhance adherence and ensure that exercise becomes a sustainable habit [121].

Complementary Forms of Physical Activity: Incorporating other forms of physical activity alongside aerobic exercise can be highly beneficial, such as strength training or flexibility exercises. Strength training can improve muscle tone, support joint health, and increase muscle mass, which in turn boosts metabolism and overall physical health [122]. Flexibility exercises, such as yoga or stretching, can reduce muscle tension, improve posture, and promote relaxation, further aiding in stress management [123]. These complementary activities provide a well-rounded approach to fitness, addressing various aspects of both physical and mental well-being.

Support from Healthcare Providers: Healthcare providers play a crucial role in supporting patients by providing education on the benefits of regular exercise, helping them set realistic goals, and offering resources such as local exercise programs or online fitness platforms. Regular follow-up appointments can help monitor progress, address any challenges, and adjust exercise plans as needed. Providers can also offer motivational support and encouragement, helping patients overcome barriers to physical activity.

Long-Term Commitment and Realistic Goals: Healthcare providers should emphasize the importance of consistency and long-term commitment to an exercise routine. While immediate benefits can be noticeable, the most significant improvements in gut health and symptom management are often observed over an extended period. Patients should be educated on setting realistic goals and expectations and encouraged to celebrate small victories to stay motivated [124]. Tools such as fitness trackers, exercise logs, and regular follow-up appointments can help patients stay on track and monitor their progress.

Social Support and Peer Engagement: Support from healthcare professionals, family, and peer support groups can also play a crucial role in maintaining adherence to an exercise program [125]. Sharing experiences and challenges with others who have similar conditions can provide motivation and practical tips for managing symptoms and staying active [126]. Online forums, local support groups, and social media communities can offer a sense of camaraderie and encouragement, making the journey towards better health more engaging and less isolating [127].

In summary, for optimal benefits, IBS patients should aim for at least 150 min of moderate to vigorous aerobic exercise per week, choosing activities that are enjoyable and sustainable. A varied and flexible approach, supported by healthcare providers and enhanced by social engagement, can help patients achieve and maintain this goal, leading to significant improvements in gut health and overall well-being. By fostering a supportive environment and providing comprehensive resources, healthcare providers can help IBS patients achieve better symptom control and an improved quality of life through regular aerobic exercise. Regular physical activity not only improves gut motility and microbiota balance but also reduces stress and enhances overall quality of life, underscoring the importance of incorporating exercise into the holistic management of IBS.

### 4.3. Monitoring and Adaptation

It is also important for healthcare providers to monitor the progress of IBS patients participating in aerobic exercise and adapt the exercise program as needed [128]. Individual responses to exercise can vary significantly due to differences in fitness levels, symptom severity, and overall health status. Therefore, a personalized approach is crucial to ensure that the exercise regimen remains effective and manageable for each patient.

Initial Assessment and Baseline Measurement: Before starting an exercise program, a comprehensive initial assessment should be conducted. This includes evaluating the patient’s current physical condition, medical history, and specific IBS symptoms. Baseline measurements such as body weight, cardiovascular fitness, muscle strength, flexibility, and symptom severity provide valuable data for tailoring an exercise program and monitoring progress over time [129]. Tools like symptom diaries or standardized questionnaires can help document initial IBS symptom patterns and track changes.

The initial assessment should also consider psychological factors such as stress levels, anxiety, and depression, which are often associated with IBS [130]. Understanding these factors can help in designing a holistic exercise program that not only addresses physical fitness but also promotes mental well-being.

Regular Follow-Ups: Regular follow-ups and assessments are essential components of effective exercise monitoring. These can be scheduled bi-weekly or monthly, depending on the patient’s needs and the severity of their condition. During these follow-ups, healthcare providers can review the patient’s exercise log, discuss any challenges or barriers they are facing, and adjust the exercise plan accordingly [131].

For example, if a patient reports increased IBS symptoms following high-intensity workouts, the provider might recommend switching to lower-intensity activities or incorporating more rest days. Conversely, if a patient is not experiencing significant benefits, the intensity or frequency of exercise might be gradually increased [132].

These follow-ups also provide an opportunity to reinforce the importance of consistency and adherence to the exercise regimen. Healthcare providers can offer encouragement, celebrate progress, and provide solutions to any emerging issues [114].

Adjusting Intensity and Duration: As patients progress, their exercise capacity and tolerance levels may change, necessitating adjustments in the intensity or duration of their routines. For instance, a patient who initially started with low-intensity activities like walking might gradually increase to moderate-intensity activities such as jogging or cycling as their fitness improves.

Conversely, if a patient experiences fatigue or increased symptoms, reducing the intensity of their exercise or incorporating more rest periods can help prevent overexertion. Adjustments should be made based on ongoing assessments and patient feedback to ensure that the exercise program remains effective and manageable [133].

Healthcare providers should also educate patients about recognizing their own body’s signals, encouraging them to communicate any discomfort or adverse reactions promptly. This proactive approach helps in making timely adjustments to the exercise plan [134].

Individualizing Exercise Plans: Tailoring the exercise program to meet the specific needs and preferences of each patient is essential for maintaining motivation and adherence. Some patients may prefer outdoor activities like hiking or biking, while others might enjoy structured classes like aerobics or dance. Incorporating patient preferences ensures that the exercise regimen is not only effective but also enjoyable, increasing the likelihood of long-term adherence [135].

Individualized exercise plans should also consider the patient’s lifestyle, work schedule, and personal commitments. Flexible scheduling options, such as early morning or evening workouts, can help patients seamlessly integrate exercise into their daily routines [136].

Utilizing Technology: Technology can play a supportive role in monitoring and adapting exercise programs. Wearable fitness trackers and smartphone apps can provide real-time data on activity levels, heart rate, and other health metrics. These tools allow patients to monitor their progress and share data with their healthcare providers, facilitating timely adjustments to their exercise plans [137].

Additionally, virtual consultations and telehealth services can provide ongoing support and guidance, especially for patients who may have difficulty attending in-person appointments [138]. Online platforms can offer exercise videos, virtual classes, and interactive forums where patients can share experiences and receive peer support.

Addressing Barriers and Providing Support: During follow-ups, it is important to address any barriers to exercise that patients may encounter, such as a lack of time, motivation, or access to facilities. Providing practical solutions, such as home-based exercise routines or recommending local community resources, can help overcome these obstacles [139].

Encouraging patients to set realistic goals and celebrating their achievements, no matter how small, can also boost their confidence and commitment to the program. Healthcare providers can use motivational interviewing techniques to help patients identify and overcome barriers, fostering a positive attitude towards exercise [140].

Holistic Approach: Monitoring and adaptation should be part of a holistic approach that considers the patient’s overall well-being. This includes managing stress, ensuring proper nutrition, and getting adequate sleep, all of which can influence the effectiveness of the exercise regimen [141].

Collaborating with other healthcare professionals, such as dietitians, psychologists, and physiotherapists, can provide comprehensive care that addresses all aspects of the patient’s health. For example, dietary modifications to support gut health, stress management techniques, and physical therapy for any underlying musculoskeletal issues can enhance the overall benefits of the exercise program [142].

In conclusion, monitoring and adapting the exercise programs of IBS patients are crucial for optimizing their benefits. Regular follow-ups, individualized adjustments, and the use of technology can help tailor the exercise regimen to each patient’s unique needs and preferences. By addressing barriers and providing continuous support, healthcare providers can enhance adherence [118] and ensure that patients achieve significant improvements in their IBS symptoms and overall quality of life.

This dynamic and responsive approach is key to the successful integration of aerobic exercise into the comprehensive management of IBS [143]. Through personalized care and continuous monitoring, healthcare providers can empower patients to take an active role in their health, leading to the more effective management of their condition and improved long-term outcomes [144].

## 5. Future Directions

Further large-scale, randomized controlled trials are necessary to validate the findings from preliminary studies and to understand the long-term effects of exercise on the gut microbiota and IBS symptoms. These studies should aim to identify the most effective types, intensities, and durations of exercise for various subgroups of IBS patients [145]. Moreover, understanding the role of gender, age, and other demographic factors in the response to exercise could help tailor interventions more precisely. For example, older adults or those with comorbid conditions may respond differently to exercise regimens compared to younger, healthier individuals [146].

Additionally, exploring the interaction between exercise, diet, and the gut microbiota could provide comprehensive insights into the management of IBS. Studies that combine exercise interventions with dietary modifications, such as an increased fiber intake or probiotics, could reveal synergistic effects on gut health and symptom relief [147]. The role of specific dietary patterns, such as low FODMAP diets, in conjunction with exercise, warrants further investigation. Combining these approaches could lead to more effective, holistic treatment plans that address both the dietary and physical activity aspects of IBS management [148].

Personalized exercise programs tailored to the individual’s gut microbiota profile and symptomatology may represent the future of IBS treatment. Advances in microbiome research and technology, such as metagenomic sequencing, could enable the development of customized exercise plans based on an individual’s unique gut microbial composition [149]. This personalized approach could optimize therapeutic outcomes by targeting the specific needs of each patient. Such precision medicine approaches are likely to become increasingly feasible as technology advances and costs decrease.

Incorporating patient feedback and experiences into the design and adjustment of exercise programs is also crucial [150]. Qualitative research methods, such as patient interviews and focus groups, can provide valuable insights into the barriers to and facilitators of exercise adherence in IBS patients. This patient-centered approach can help refine exercise recommendations, making them more practical and sustainable. Understanding patient perspectives on exercise, including preferences, motivations, and perceived obstacles, can enhance the design of intervention strategies that are both effective and enjoyable [151].

Collaborative efforts between researchers, clinicians, and patients are essential to translate scientific findings into clinical practice effectively [152]. Interdisciplinary research teams, including gastroenterologists, exercise physiologists, dietitians, and psychologists, can work together to develop comprehensive treatment protocols that address the multifaceted nature of IBS. These teams can also facilitate the integration of exercise programs into standard care, ensuring that patients receive consistent and coordinated support [153].

Lastly, public health initiatives that promote physical activity and educate about its benefits for gut health could play a significant role in the broader management of IBS. Community-based programs and digital health platforms can increase the accessibility of tailored exercise regimens and support networks for IBS patients. Public health campaigns can raise awareness about the importance of physical activity for gut health, potentially encouraging more individuals with IBS to engage in regular exercise [154].

Digital health platforms, such as mobile apps and online communities, can provide resources, tracking tools, and social support, making it easier for patients to adhere to their exercise programs. These platforms can also facilitate remote monitoring and consultations, offering convenience and continuous support for patients [155].

In conclusion, while the current body of research underscores the potential of exercise to improve the gut microbiota and alleviate IBS symptoms, ongoing studies are crucial to fully harness this potential. By focusing on personalized, integrative approaches and fostering collaborative research, the medical community can significantly advance the management and treatment of IBS, ultimately enhancing the quality of life for those affected by this condition [156].

Emphasizing the need for further research, particularly large-scale and long-term studies, will help to solidify the evidence base for exercise as a therapeutic intervention for IBS. Personalized exercise programs, informed by cutting-edge microbiome research, have the potential to transform IBS management. By integrating patient feedback and adopting a multidisciplinary approach, healthcare providers can develop effective, sustainable exercise regimens that address the unique needs of each patient [157].

Public health initiatives and digital health innovations will also play a key role in promoting exercise and supporting patients. Through these combined efforts, the goal of improved symptom management and enhanced quality of life for IBS patients can become a reality [158].

## 6. Conclusions

The impact of exercise on the gut microbiota in IBS patients is a promising area of research with significant clinical implications. Aerobic exercise, resistance training, and combined training each offer unique benefits for gut health and symptom management in IBS. Future research should focus on elucidating the specific mechanisms through which different types and intensities of exercise influence the gut microbiota and identifying optimal exercise regimens for individual IBS patients. Integrating exercise into standard IBS treatment protocols could enhance therapeutic outcomes and improve the quality of life for IBS patients.
